# A comparison of anatomical and dosimetric variations in the first 15 fractions, and between fractions 16 and 25, of intensity‐modulated radiotherapy for nasopharyngeal carcinoma*

**DOI:** 10.1120/jacmp.v14i6.4424

**Published:** 2013-11-04

**Authors:** Haihua Yang, Yu Tu, Wei Wang, Wei Hu, Weijun Ding, Changhui Yu, Chao Zhou

**Affiliations:** ^1^ Department of Radiation Oncology Taizhou Hospital Wenzhou Medical College Taizhou Zhejiang China; ^2^ Laboratory of Cellular and Molecular Radiation Oncology Taizhou Hospital Wenzhou Medical College Taizhou Zhejiang China; ^3^ School of Radiation Medicine and Protection Medical College of Soochow University Jiangsu Provincial Key Laboratory of Radiation Medicine and Protection Suzhou Jiansu China

**Keywords:** replans, nasopharyngeal carcinoma, intensity‐modulated radiotherapy

## Abstract

The purpose of this study was to compare anatomical and dosimetric variations in first 15 fractions, and between fractions 16 and 25, during intensity‐modulated radiotherapy (IMRT) for nasopharyngeal carcinoma (NPC). Twenty‐three NPC patients who received IMRT in 33 fractions were enrolled. Each patient had two repeat computed tomography (CT) scans before the 16th and 25th fraction. Hybrid IMRT plans were generated to evaluate the dosimetric changes. There was a significant decrease of the transverse diameter of nasopharyngeal and neck as well as gross tumor volume (GTV) in the primary nasopharyngeal carcinoma (GTVnx) and involved lymph nodes (GTVnd) during the first 15 fractions, and between fraction 16 and 25 (p<0.05). Consequently, there was a significant reduction of the percentage of the volume receiving the prescribed dose $\left(V_{100}\right)$ of CTV1 and GTVnd, which was more prominent after the first 15 fractions treatment compared to that between fraction 16 and 25 (p<0.05). Additionally, there was a significant increase in the mean dose (Dmean) and percentage of volume receiving $\geq 30\;\text{Gy}\;(V_{30})$ to the bilateral parotid in the first 15 fractions (p<0.05), but not between fraction 16 and 25. While the maximum dose to the spinal cord was significantly increased both in the first 15 fractions, and between fraction 16 and 25 (p<0.05), the increase of the percent of spinal cord volume receiving $\geq 40\;\text{Gy}\;(V_{40})$ was significantly higher in the first 15 fractions compared to that between fraction 16and25(p<0.05). Based on the dose constraint criterion in the RTOG0225 protocol, a total 39.1%(9/23) of phantom plan 1 (generated by applying the beam configurations of the original IMRT treatment plan to the anatomy of the second CT scan) and 17.4%(4/23) of phantom 2 (generated by applying the beam configurations of the replan 1 to the anatomy of the third CT scan) were out of limit for the dose to the normal critical structures. In conclusion, our data indicated that anatomic changes resulted in more predominant dosimetric effects in the first 15 fractions, and between fractions 16 and 25, of IMRT.

PACS number: 87.53.Bn, 87.55.de, 87.55.Qr

## I. INTRODUCTION

Nasopharyngeal carcinoma (NPC) is common among Asians, especially the Southern Chinese.1(1) Radiation therapy with or without chemotherapy is the definitive treatment for NPC.^2^, ^3^, ^4^ In external beam radiotherapy, treatment has always aimed at administering an adequate dose coverage to the entire tumor volume while protecting the surrounding normal tissues. The relationship between the planning dose constraints and the resultant dose distributions depends on several factors, especially variations in the anatomic relationship between the tumor and sensitive structures. Some patients receiving radiation therapy (RT) to the head and neck will have significant anatomic changes during their treatment course, including shrinking primary tumors or nodal masses, resolving postoperative changes/edema, and changes in overall body habitus/weight loss.^5^, ^6^, ^7^, ^8^, ^9^, ^10^, ^11^, ^12^ These variables could theoretically cause deviations in radiation dose delivery from the initial treatment plan, especially the highly conformal treatment approaches,^13^, ^14^, ^15^ such as intensity‐modulated radiotherapy (IMRT).^16^, ^17^, ^18^, ^19^, ^20^, ^21^ It has been reported that replanning by using the second CT scan with an average interval of 19±6 fractions during the course of IMRT for head and neck cancer patients significantly reduced the normal organ dose and increased the target dose coverage, compared with using the original plan on the new anatomy.^(17)^ Our previous studies implicated that 50% of IMRT plans may need replanning before the 25th fraction because of the overdose to the normal sensitive structures.^(^ ^20^ ^,^ ^22^ ^)^ Recently, Zhao et al.^(23)^ conducted a retrospective study to demonstrate that the IMRT replan improved the three‐year local progression‐free survival for patients who had American Joint Committee on Cancer (AJCC) stage higher than T3 (T3, 4Nx) and eased the after effects for patients who had large lymph nodes (AJCC stage TxN2,3). However, there is a lack of studies that compare the effects of different time periods of repeat CT scans and replans on conformality and dose distributions during IMRT treatment, which may be helpful to decide the optimal timing of replans during IMRT. We conducted the present study to compare anatomical and dosimetric variations in the first 15 fractions, and between fraction 16 and 25, during the course of IMRT for NPC patients.

## II. MATERIALS AND METHODS

### A. Patient characteristics

Twenty‐three consecutive patients, who were newly diagnosed with nasopharyngeal carcinomas without evidence of distant metastasis and were treated definitively with IMRT in 33 fractions alone or with concomitant chemotherapy between November 2008 and December 2009, were selected for this study. A simulation computed‐tomography (CT) scan was acquired for each patient before the start of the treatment and before the 16th and 25 th fraction during the course of treatment. The patient selection process included a complete medical history, physical examination, direct flexible fiber‐optic endoscopic examination, pathologic diagnosis, complete blood counts, liver and renal function tests, chest X‐ray, type B ultrasound of the abdomen and cervix, contrast‐enhanced CT and magnetic resonance imaging (MRI) scans of head and neck region, and whole‐body bone scans. The International Union Against Cancer/American Joint Committee on Cancer's 2002 staging classification was used for disease staging.^(24)^ All patients provided written informed consent before enrollment. The pretreatment and treatment characteristics of the patients are listed in Table 1.

**Table 1:** Demographic and baseline characteristics of patients

*Characteristics*	*Value*
Patients, n	23
Median age, years (range)	50(28–71)
Sex, n (%)	
Male	16 (70)
Female	7 ( 30)
KPS, n (%)	
100	3 (13)
90	7 (30)
80	11 (48)
70	2 (9)
T‐category, n (%)	
1	6 (26)
2	9 (39)
3	4 (17)
4	4 (17)
N‐category, n (%)	
0	5 (22)
1	11 (49)
2	5 (22)
3	2 (9)
Stage‐group, n (%)	
I	3 (13)
II	9 (39)
III	5 (22)
IVa/b	6 (26)
Concurrent chemotherapy, n (%)	
Yes	14 (61)
No	9 (39)

KPS = Karnofsky performance status score

### B. CT image and study plan

All patients underwent immobilization with a head and shoulder mask and a head and shoulder board before the CT scan. All CT scans had 2.5 mm slice thickness through the head and neck region from the skull vertex to 2 cm below the clavicles. The three CT scans of each patient were performed in the same position, orientation, and the same external reference markers by the same technician. In order to make sure the isocenter and the external reference markers remain the same, the immobilization device has not been refashioned.

The following CT scans and plans were studied:

1. The initial treatment CT scan (first CT) was performed within two days of the treatment. The original IMRT plan (initial plan) was generated based on this initial CT scan.
2. The second treatment CT scan (second CT) was performed before the 16th fraction of IMRT in all patients. The first replan (replan 1) was generated based on this second CT scan.
3. The third treatment CT scan (third CT) was performed before the 25 th fraction of IMRT. The second replan (replan 2) was generated based on this third CT scan.
4. Phantom plan 1 was generated by applying the beam configurations (including the intensity profile of each beam) of the original IMRT treatment plan to the anatomy of the second CT scan.
5. Phantom plan 2 was generated by applying the beam configurations (including the intensity profile of each beam) of the replan 1 to the anatomy of the third CT scan.

For each patient, in order to minimize the errors due to the loosening of the immobilization device between the three CT scan simulations, the spatial relationship of the isocenters of the three CT scans was established by using CT‐CT fusion based on boney landmarks for each patient. Repeat CT scans were fused to the first CT. The phantom plans were generated by using the Quality Assessment Center of the CORVUS 6.3 inverse planning system (version 6.3, NOMOS Corporation, Cranberry Township, PA) after the phantom shift was eliminated (unity of dose calculation reference point). The phantom plans were generated by the same physicians in order to minimize the delineation variability among observers. After recontouring, the initial treatment plan was mapped to the second CT scans and the replan was mapped to the third CT scans with the same beam configurations. The phantom plans in this study represented the actual dose distributing that would have been delivered had the patient not been replanned before the 16th and 25th fraction of IMRT.

### C. Delineation of target volume and treatment planning delivery

The gross tumor volumes (GTVs) included the primary nasopharyngeal tumor (GTVnx) and involved lymph nodes (GTVnd), as shown by clinical information and endoscopic and radiologic examinations (including CT and MRI). The clinical target volume (CTV) included the high‐risk regions (CTV1) and the low‐risk regions (CTV2). For the initial treatment plan, MR images were fused to the simulation CT images using the CORVUS 6.3 inverse planning system to help the delineation of target volumes. The nasopharyngeal regions and upper neck IMRT plans were generated and approved for each patient using the CORVUS 6.3 inverse planning system. The IMRT plan was delivered using a sequential helical tomotherapy technique with special MLC (MINIC; NOMOS), whereas the lower neck and the supraclavicular regions were treated with a conventional anterior posterior (AP) field, so the volume and dosimetric comparisons for these regions were excluded. The IMRT field was matched with the AP field with a split‐beam technique.

The planning target volume (PTV) and the planning organs‐at‐risk volume (PRV) were defined as having an additional 3 mm and 2 mm margin to compensate for the variability of treatment setup and internal organ motion, respectively. A total of 70–76 Gy (2.12–2.3 Gy/fraction), 66–70 Gy (2.0–2.12 Gy/fraction), 60–66 Gy (1.8–2.0 Gy/fraction), and 56–60 Gy (1.7–1.8 Gy/fraction) were delivered to PTVs of the GTVnx, GTVnd, CTV1, and CTV2, respectively, in 33 fractions with simultaneous integrated boost. The lower neck and supraclavicular regions received 50–60 Gy at 2.0 Gy/fraction/day with conventional radiotherapy.

Two replans were generated at the 16th and 25th fraction of IMRT and used to complete the planned course of treatment. Based on dose constraint criteria in the RTOG 0225 protocol, the replans were generated on the new simulation CT scans. For each patient, all target volumes and normal structures were manually outlined slice by slice on the simulation CT images by the same attending physician. Attempts were made to maintain the original CTVs with modification that adapt to the changes in anatomic structure displayed in the repeat CT scans. GTVs were recontoured according to the shrinkage or/and distortion of primary tumor or lymph nodes shown in the new CT scans. Normal structures and critical organs were recontoured the same as the original plan.

### D. Anatomical comparison

The transverse diameter of the nasopharyngeal level (d1) represents the distance between the intersection points on both sides of skin edges, at the level of the odontoid process. It is a posterior marginal connection for the bilateral mandibular angle. Transverse diameter of the neck level (d2) represents the distance between the intersection points on both sides of the skin edges, at the level of the lower edge of cervical vertebra 3. It is a horizontal line at the front of the vertebral body.^(22)^ The transverse diameters of the nasopharynx and neck were compared between the first, second, and third CT scans. Target volume and sensitive structure volumes were also compared between these three CT scans.

### E. Dosimetric comparison

Dose‐volume histograms (DVHs) were calculated for target volumes and normal structures for each IMRT plan. The phantom plan 1 was compared to the original plan to investigate the effects of anatomic changes on dosimetric outcomes during the first half of the treatment. The phantom plan 2 was compared to replan 1 to investigate the effects of anatomic changes on dosimetric outcomes between fractions 16 and 25 of treatment.

### F. Statistical analysis

The Statistical Package for the Social Sciences (SPSS for Windows 13.0, SPSS Inc., Chicago, IL) was used for statistical analysis. Descriptive statistics were calculated to characterize the volume and dose parameters. Categorical variables are presented as frequencies and percentages. Chi‐square test was used for categorical variables. Comparisons between the two paired CT volume and dosimetric parameters of the original plan vs. phantom plan 1 and the replan vs. phantom plan 2 were analyzed using the paired samples t‐test. ANOVA was used to compare means between the three groups. All p‐values are two‐sided. A p‐value ≤0.05 was considered statistically significant.

## III. RESULTS

### A. Anatomical comparison

#### A.1 Transverse diameters of the nasopharynx and the neck

**Figure 1:**
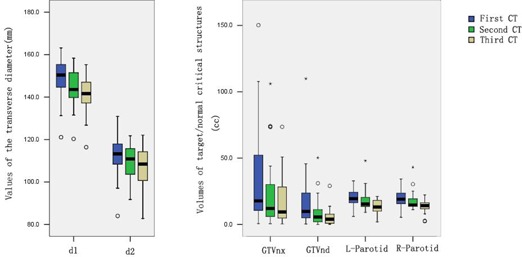
Average diameters and volumes of the targets and the parotid glands in three CT images of 23 cases of nasopharyngeal carcinoma. d1, d2, and the target volume (GTVnx and GTVnd) decreased gradually in three CT scans. The volumes of the two parotid glands decreased in third CT scan compared to the first and second CT scan. Boxes represent the 25th and 75th percentiles; horizontal bars within each box represent the median values; circles denote outliers; asterisks denote the extreme value. d1 = transverse diameter of the nasopharyngeal level; d2 = transverse diameter of the neck level; GTVnx = gross tumor volumes of primary nasopharyngeal tumor; GTVnd = gross tumor volumes involved lymph nodes; L‐parotid = Left parotid; R‐parotid = Right parotid.

Anatomic changes were determined based on the original CT scan and two repeat CT scans. As shown in Fig. 1 and Table 2, the transverse diameters of the nasopharynx (d1) and the neck (d2) decreased over the course of treatment (p<0.05). The average transverse diameters of the nasopharynx in the first, second, and third CT scan were 148.8±10.0mm,144.2±9.1mm,and140.9±8.9mm, respectively. The average transverse diameters of the neck in the first, second, and third CT scan were 112.4±10.5mm,109.8±8.1mm,and106.6±9.1mm, respectively.

**Table 2:** Percentage of anatomical changes in three CT images (n=23)

	$1^{\text{st}}$ CT–$2^{\text{nd}}$ *CT*			*2nd* $\text{CT}-3^{\text{rd}}\text{CT}$			$1^{\text{ST}}\text{CT}-3^{\text{rd}}\text{CT}$			
	*Percentage changes (%)*	*95% CI of changes*	$P_{1}$	*Percentage changes (%)*	*95% CI of changes*	$P_{2}$	*Percentage changes (%)*	*95% CI of changes*	$P_{3}$	$P_{4}$
d1	3.2±2.8	1.9−4.4	0.000	2.2±1.5	1.5−2.9	0.000	5.3±3.2	4.0−6.7	0.000	0.190
d2	2.3±4.4	0.4−4.2	0.017	2.8±3.5	1.3−4.3	0.001	5.2±5.2	2.9−7.4	0.000	0.710
CTV1	2.0±6	−0.6–4.5	0.135	0.0±6.5	−2.8–2.8	0.988	2.0±4.9	−0.1–4.1	0.066	0.433
GTVnx	34.6±53.0	11.6−57.6	0.005	17.3±39.1	0.3−34.0	0.047	51.9±62.7	24.7−78.8	0.001	0.230
GTVnd^a^	45.9±72.3	10.3−82.1	0.015	24.8±33.6	8.3−41.3	0.006	70.7±99.6	21.2–120	0.008	0.100
Volume of left parotid gland	7.1±45.7	−12.7–26.9	0.460	24.9±47.2	4.6−45.2	0.019	32.0±24.9	21.3−42.6	0.000	0.350
Volume of right parotid gland	10.2±43.4	−8.7–28.6	0.273	20.9±40.3	3.6−38.3	0.021	31.1±25.0	20.4−41.8	0.000	0.520

a Five cases were node‐negative.

CI = confidence interval; d1 = transverse diameter of the nasopharyngeal level; d2 = transverse diameter of the neck level; GTVnx = gross tumor volumes of primary nasopharyngeal tumor; GTVnd = gross tumor volumes involved lymph nodes; CTV1 = high‐risk regions of clinical target volume; 1st CT = performed within two days before the treatment; 2nd CT = performed before the 16th fraction; 3rd CT = performed before the 25th fraction; P1 = p‐value for the difference between 1st CT and 2nd CT; P2 = p‐value for the difference between 2nd CT and 3rd CT; P3 = p‐value for the 1st CT and 3rd CT. P4 = p‐value for the comparison of change between 1st CT and 2nd CT, and that between 2nd CT and 3rd CT.

#### A.2 Volumes of target

Figure 1 compares the volumes of the target and Table 2 compares the percentage changes of the volumes of the target in the three CT scans. GTVnx and GTVnd, but not CTV1, significantly decreased after 15 fractions of treatment by comparing the initial and second CT scans (p<0.05). The similar outcome was revealed when we compared GTVnx, GTVnd, and CTV1 between the second and third CT scans, or between the first and third CT scans.

#### A.3 Volumes of parotid glands

In our study, we treated the left and right parotid gland as two separate structures. The volumes of the left and right parotid glands decreased over the course of IMRT (Fig. 1). There were no significant differences in the volumes of the bilateral parotid glands in the first 15 fractions (p>0.05). However, as shown in Table 2, the average volumes of the left and right parotid glands were decreased significantly between fractions 16 and 25 (p<0.05).

### B. Dosimetric comparison

#### B.1 Target doses

**Figure 2:**
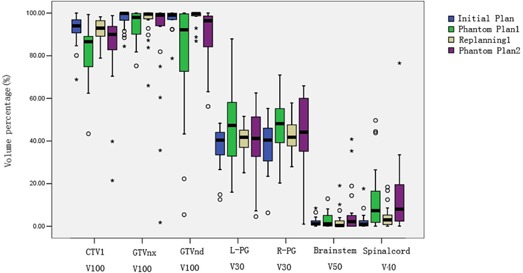
Changes of volume percentage of the target and normal structure receiving certain doses in the four plans. The $V_{100}$ of CTV1, GTVnd significantly decreased in phantom plan 1 and phantom plan 2, compared to the initial plan and replan. There was a significant increase in the mean dose and $V_{30}$ of the bilateral parotid in phantom plan compared to the initial plan. The $V_{50}$ of the brain stem significantly increased between fraction 16 and 25. Boxes represent the 25th and 75th percentiles; horizontal bars within each box represent the median values; circles denote outliers; asterisks denote the extreme value. CTV1 $V_{100}$ = percent of high‐risk regions of clinical target volume receiving the total prescribed dose; CTVnx $V_{100}$ = percent of gross tumor volumes of primary nasopharyngeal tumor receiving the total prescribed dose; CTVnd $V_{100}$ = percent of gross tumor volumes involved lymph nodes receiving the total prescribed dose; L‐PG $V_{30}$ = percent of left parotid gland volume receiving 30 Gy; R‐PG $V_{30}$ = percent of right parotid gland volume receiving 30 Gy; BS $V_{50}$ = percent of brain stem volume receiving 50 Gy; SC $V_{40}$ = percent of spinal cord volume receiving 40 Gy.

The percentage of the volume receiving the prescribed dose $\left(V_{100}\right)$ of CTV1, GTVnx, and GTVnd were compared between the initial plan vs. phantom plan 1 and replan 1 vs. phantom plan 2. The $V_{100}$ of CTV1 and GTVnd were significantly lower in the two phantom plans than in the initial plan (p<0.05) (Fig. 2). Importantly, the changes of the $V_{100}$ of CTV1 and GTVnd were significantly higher in the first 15 fractions, compared to that between fraction 16 and 25 (p<0.05).

#### B.2 Parotid gland doses

**Figure 3:**
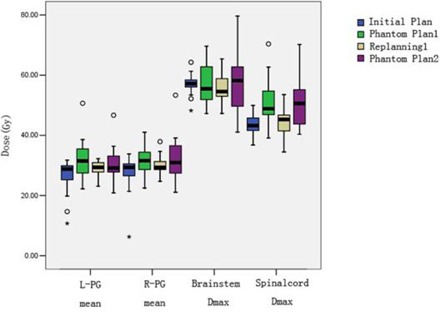
Changes of doses to normal structure in the four plans. The doses to two parotid glands and spinal cord significantly increased in phantom plan 1, compared to initial plan. The doses to spinal cord also significantly increased in phantom 2 compared to replan 1. Boxes represent the 25th and 75th percentiles; horizontal bars within each box represent the median values; circles denote outliers; asterisks denote the extreme value. L‐PG Dmean = mean dose to left parotid gland; R‐PG Dmean = mean dose to right parotid gland; BS Dmax = maximum dose to brain stem; SC Dmax = maximum dose to spinal cord.

In the comparison between the initial plan and phantom plan 1 for the left parotid gland doses, there was a significant increase in the mean dose (Dmean) and percentage of volume receiving $\geq 30\;\text{Gy}\;(V_{30})$ to the bilateral parotid in the first 15 fractions (p<0.05). However, these differences between replan 1 and phantom plan 2 did not reach statistical significance (Fig. 3, Table 3).

**Table 3:** Percentage of dose changes of the target/normal critical structures (n=23)

	*Initial Plan – Phantom Plan 1*			*Replan 1 – Phantom Plan 2*			
	*Percentage changes*	*95% Cl of changes*	$P_{1}$	*Percentage changes*	*95% Cl of changes*	$P_{2}$	$P_{3}$
Targets							
$\text{CTVl}\;V_{100}{\left(\%\right)}^{+}$	12.7±16.3	5.5−19.7	0.001	8.9±18.6	0.9−16.9	0.032	0.029
$\text{GTVnx}\;V_{100}\left(\%\right)+$	3.0±6.1	0.3−5.6	0.280	8.0±23.5	−2.2–18.1	0.160	0.168
GTVnd $V_{100}$ (%b)^a^	20.0±28.3	5.4−34.5	0.010	9.1±13.3	2.3−15.9	0.012	0.000
Left parotid							
$D_{\text{mean}}\;{\left(\text{Gy}\right)}^{a}$	−19.2±33.5	−33.5—4.5	0.013	−4.5±19.2	−12.8–3.8	0.246	0.340
$v_{30}\;{\left(\%\right)}^{b}$	−21.6±49.1	−42.7—0.2	0.047	4.7±39.6	−12.4–21.9	0.571	0.058
Right parotid							
$D_{\text{mean}}\;(\text{Gy})$ ^b^	−10.8±8.2	−18.9–2.9	0.009	−5.8±5.3	−12.4–0.8	0.082	0.079
$v_{30}\;{\left(\%\right)}^{b}$	−32.1±45.1	−51.6–12.3	0.003	−4.3±63.9	−31.8–23.1	0.747	0.074
Brainstem							
$D_{\text{max}}\;{\left(\text{Gy}\right)}^{b}$	−0.2±11.1	−4.9–4.7	0.970	−3.5±4.2	−9.6–2.6	0.257	0.158
$v_{50}\;{\left(\%\right)}^{b}$	−45±130	−100–15	0.128	−190±425	−375–0.0	0.042	0.979
Spinal cord							
$D_{\text{max}}\;{\left(\text{Gy}\right)}^{b}$	−17.7±4.7	−24.2—11.3	0.000	−13.6±3.6	−19.4—7.8	0.000	0.155
$v_{40}\;{\left(\%\right)}^{b}$	−450±554	−692—208	0.001	−392±663	−679—108	0.009	0.000

a Decrease in phantom plan.

b Increase in phantom plan.

V100 = percent of volume receiving the total prescribed dose; D_max_ = maximum dose; D_mean_ = mean dose; V_50_, V_40_, and V_30_ = percent of volume receiving . 50 Gy, . 40 Gy, and .30Gy, respectively; Phantom plan 1 = a phantom plan was generated for each patient by applying the beam configurations of the initial plan to the phantom before the 16th fraction; Replan1 = replan before the 16th fraction; Phantom plan 2 = a phantom plan was generated for each patient by applying the beam configurations of the replan before the 16th fraction to the phantom before the 25th fraction. P_1_ = p‐value for the difference between Initial plan and Phantom plan 1; P_2_ = p‐value for the difference between Replan 1 and Phantom plan 2; P_3_ = p‐value for the comparison of change between Initial plan and Phantom plan 1, and that between Replan 1 and Phantom plan 2.

#### B.3 Serial‐sensitive structure doses

While the maximum dose (Dmax) to the spinal cord was significantly increased both in the first 15 fractions, and between fraction 16 and 25 (p<0.05) (Fig. 3), the increase of the percent of spinal cord volume receiving $\geq 40\;\text{Gy}\;(V_{40})$ was significantly higher in the first 15 fractions compared to that between fraction 16 and 25 (p<0.05). The percent of the volume of the brain stem receiving $\geq 50\;\text{Gy}\;(V_{50})$ significantly increased between fraction 16 and 25, but not during the first 15 fractions (p<0.05) (Table 3).

### C. Plan conformity

Based on the dose constraint criterion about brain stem and spinal cord and parotid gland in the RTOG0225 protocol,^(25)^ a total 39.1% of phantom plan 1 (9/23) and 17.4% (4/23) of phantom 2 were out of limit for the dose to the normal critical structures (Table 4).

**Table 4:** Numbers of plans with dose contributions exceeding normal critical structures criteria (n=23)

	*Initial Plan n (%)*	*Phantom Plan 1 n (%)*	$P^{1}$	*Replan 1 n (%)*	*Phantom Plan 2 n (%)*	$P^{2}$
$\text{Both parotid glands mean dose}>26\;\text{Gy and}\;V_{30}>50\%$	0 (0.0%)	5 (21.7%)	0.049	0 (0.0%)	2 (8.7%)	0.489
$\text{Dose of brain stem}>54\;\text{Gy and}\;V_{60}>1\%$	0 (0.0%)	3 (13.0%)	0.233	0 (0.0%)	2 (8.7%)	0.489
$\text{Dose of spinal cord}>45\;\text{Gy and}\;V_{50}>1\;{\text{cm}}^{3}$	0 (0.0%)	5 (21.7%)	0.022	0 (0.0%)	1 (4.4%)	1.000
With one of the above four conditions	0 (0.0%)	9 (39.1%)	<0.001	0 (0.0%)	4 (17.4%)	0.109

## IV. DISCUSSION

In the present study, we conducted a prospective study to quantify the anatomic changes and their dosimetric effects during the first 15 fractions, and between fraction 16 and 25, of IMRT for patients with NPC. Our study showed there were significant anatomic changes after the treatment of first 15 fractions, and between fraction 16 and 25 of IMRT, based on repeat CT scans. Dosimetric effect of changes in anatomy was more predominant in the first 15 fractions compared to that between fractions 16 and 25 of IMRT, not only in the coverage of the target but also of the critical structures, except for brain stem.

Many patients with head and neck cancer have tumor shrinkage and/or weight loss during the course of radiotherapy. Additionally, volumetric changes and spatial variability have often resulted in dosimetric effects.^(^ ^17^, ^18^, ^19^, ^20^, ^21^ ^,^ ^26^, ^27^, ^28^, ^29^ ^)^ In our study, there was significant decrease of GTVnx and GTVnd during the treatment of IMRT for patients with NPC. Due to the anatomical modifications, the target doses also decreased in the phantom plans. The $V_{100}$ of CTV1 and GTVnd were significantly lower in the two phantom plans than in the initial plan. Our results were consistent with the previous report that the doses to 95% of the planning target volumes of the gross tumor volume and the clinical target volume were reduced during the course of IMRT for patients with head and neck cancer.^(17)^ However, it was reported in another study that the anatomical changes had no effect on tumor dose coverage in patients with head and neck cancer.^(26)^

Although the necessity of repeat CTs and replans during IMRT has been increasingly realized, there is limited data about the comparison of anatomical and dosimetric variations at different time period during IMRT for cancer patients. Our findings showed the dose reductions of CTV1 and GTVnd were more prominent during the 15 fractions treatment, compared to that between fraction 16 and 25. These findings are in agreement with the previous report that the most significant volumetric changes and dosimetric alterations in the tumor volumes and organs at risk occur by Week 2 of radiotherapy during a course of induction chemotherapy followed by chemoradiotherapy with intensity‐modulated radiation therapy for head and neck cancer patients.^(30)^ However, the dose of GTVnx in our study was not significantly decreased. This may be due to the minor reduction in the volume of GTVnx, with smaller displacement of nasopharyngeal anatomy.

A number of studies have reported that the parotid glands underwent volume reduction^(^ ^10^, ^11^, ^12^ ^,^ ^16^, ^17^, ^18^, ^19^, ^20^, ^21^ ^,^ ^29^ ^)^ and a median translation during IMRT.^(^ ^10^ ^,^ ^12^ ^)^ In the present study, both the left and right parotid glands showed significant volume shrinkage before the 25th fraction. Surprisingly, we observed that the parotid volume underwent a larger reduction between fractions 16 and 25 than in the first 15 fractions. This may be related with the radiation‐induced parotid edema in the first 15 fractions. These results conflict with a previous report that demonstrated volume loss in the parotid throughout the treatment course.^(16)^ However, while there was significant change of parotid gland volume between fractions 16 and 25, the dose to the parotids did not show significant change. Both tumor reduction and neck lymph node shrinkage due to radiotherapy or chemoradiotherapy tended to be greater in the first 15 fractions of treatment. Enlargement of the lymph nodes near the parotid glands and the primary tumor pushed the parotid gland outward before treatment. So the portion of parotid glands might have fallen into the GTV area after radiotherapy if the treatment plan was not modified in the first 15 fractions.

Spinal cord and brainstem are the most important of the critical structures considered in head and neck radiotherapy. In a retrospective study of 13 patients with head and neck cancer, the spinal cord Dmax increased in all patients by 0.2–15.4 Gy, and the brainstem Dmax increased in 85% of patients by 0.6–8.1 Gy without replanning (the interval time between the two CT scans was39±11 days).^(17)^ In our previous pilot study of 28 NPC patients who received the second CT scan before the 25th fraction, the spinal cord Dmax and brain stem Dmax also decreased by 1.42–8.58 Gy and −0.31−8.8Gy, respectively, in replans.^(20)^ Those studies only performed a single repeat planning CT scan during the treatment course. In the present study, we performed two repeat CT scans in the first 15 fractions and between fractions 16 and 25 to compare the differences of dosimetric changes between the two phases. We found that the doses to spinal cord increased not only in the first 15 fractions, but also between fractions 16 and 25 of treatment in the unmodified plans. Additionally, the percent of the volume of the brain stem receiving ≥50Gy significantly increased between fraction 16 and 25. The dosimetric fluctuation of critical structures depends on several parameters, such as the spatial displacement itself, the proximity of the critical structures to the target volume, the shape of the dose distribution, and proximal dose gradients. Because the spinal cord is in a horseshoe‐shaped structure, the dose fluctuations of the spinal cord could be attributed not only to anterior‐posterior displacement, but also to lateral displacement. Therefore, the dose changes in the spinal cord were similar in the first 15 fractions, and between fractions 16 and 25. However, the dose to brain stem showed significant changes only between fractions 16 and 25, not in the first half. This could be mainly because the changes in brain stem dose are only attributable to anterior–posterior displacement. Brainstem is also immediately adjacent to the high‐dose region, so tumor reduction after radiation would relax the position of the brain stem and make it move into the high‐dose region formerly occupied by the tumor. Therefore, the brainstem might have experienced slight special displacement, resulting in a large dosimetric effect.

In the present study, in the processes of repeat CT scans, the immobilization device, the isocenter, and the external reference markers remain the same to decrease the errors. In order to further minimize the errors due to the loosening of the immobilizing mask, we fused the two replan images to the original image using rigid bony registration. It has been recently reported that rigid boney registration itself may lead to errors arising out of patient movement in the shell.^(23)^ Therefore, future studies using better deformable registration algorithm will help to validate the criteria for repeat CT imaging and IMRT replanning in NPC patients undergoing radiotherapy.^(31)^

## V. CONCLUSIONS

The results of the present study indicated repeat CT imaging and replanning is recommended to ensure adequate dose to the target volumes and safe doses to critical normal structures, and to maximize the therapeutic effects during the entire IMRT treatment course for NPC patients. Dosimetric changes resulting from anatomic alterations were more predominant in the first 15 fractions than that between fractions 16 and 25 of IMRT treatment for patients with NPC. Further studies are needed to determine whether IMRT replanning at midcourse is superior over replanning at a latter course.

## ACKNOWLEDGMENTS

This study was supported by Zhejiang Provincial Medical and Health Science Foundation of China (2008B198 and 2009A221) and the Priority Academic Program Development of Jiangsu Higher Education Institutions (PAPD). We acknowledge Mrs. Sandra Berger from University of Kentucky for manuscript editing.

## Supporting information

Supplementary Material

Supplementary Material
