# Supplementary material for: A comparison of anatomical and dosimetric variations in the first 15 fractions, and between fractions 16 and 25, of intensity‐modulated radiotherapy for nasopharyngeal carcinoma*
Source: J Appl Clin Med Phys. 2013 Nov 4;14(6):1–13. doi: 10.1120/jacmp.v14i6.4424 (PMC5714644; doi:10.1120/jacmp.v14i6.4424)
Supplement: Supplementary file 1 — Supplementary Material [file ACM2-14-001a-s001.docx]

**VII-Appendix**

The algorithm to calculate profile is as follows:

1. PDDs are measured for a number of square fields along the central beam axis, for open fields and 45º wedge and for both energies 6 and 18 MV separately. The PDDs are tabulated for desired interval (e.g., 0.5cm) and stored in the MATLAB program.
2. Basic beam parameters as Input data: Field size, wedge angle and blocks properties (e.g., thickness, location and size)
3. For open symmetric field, the equivalent square (S_eq_, x_0_, 0) is calculated for points on the profile (Eq.5).
4. PDD(S_eq_,x_0_,0) is interpolated from data stored at depth d.
5. According Eq.6, 7, profile is calculated.
6. The γ-index is calculated for every point. σ_in_, σ_out_, T_J_ and CF_e.diseq_ need to be changed till$\gamma\leq1$.
7. After this, CF_J_, CF_w_ and CF_b_ can be calculated then profile can be plotted for asymmetric, wedged and irregular fields.
