# Supplementary material for: A comparison of anatomical and dosimetric variations in the first 15 fractions, and between fractions 16 and 25, of intensity‐modulated radiotherapy for nasopharyngeal carcinoma*
Source: J Appl Clin Med Phys. 2013 Nov 4;14(6):1–13. doi: 10.1120/jacmp.v14i6.4424 (PMC5714644; doi:10.1120/jacmp.v14i6.4424)
Supplement: Supplementary file 2 — Supplementary Material [file ACM2-14-001a-s002.docx]

**VII-Appendix**

In this appendix, the procedure to transition Eq. 2 from Eq.1 is explained. Using a polar system and eliminate the small area at the origin of coordinate with radius ɛ and dividing the field into eight parts regarding the origin and integrating both sides of Eq. 1, it can be written as:

$$\left(\begin{matrix} \int_{0}^{\tan^{-1}(\frac{Y1}{X1})} \int_{\varepsilon}^{\frac{X1}{\cos(\theta)}} \frac{drd\theta}{r}+\int_{\tan^{-1}(\frac{Y1}{X1})}^{\frac{\pi}{2}} \int_{\varepsilon}^{\frac{Y1}{\sin(\theta)}} \frac{drd\theta}{r}+\int_{0}^{\tan^{-1}(\frac{Y1}{X2})} \int_{\varepsilon}^{\frac{X2}{\cos(\theta)}} \frac{drd\theta}{r}+\int_{\tan^{-1}(\frac{Y1}{X2})}^{\frac{\pi}{2}} \int_{\varepsilon}^{\frac{Y1}{\sin(\theta)}} \frac{drd\theta}{r}+\int_{0}^{\tan^{-1}(\frac{Y2}{X2})} \int_{\varepsilon}^{\frac{X2}{\cos(\theta)}} \frac{drd\theta}{r} \\ +\int_{\tan^{-1}(\frac{Y2}{X2})}^{\frac{\pi}{2}} \int_{\varepsilon}^{\frac{Y2}{\sin(\theta)}} \frac{drd\theta}{r}+\int_{0}^{\tan^{-1}(\frac{Y2}{X1})} \int_{\varepsilon}^{\frac{X1}{\cos(\theta)}} \frac{drd\theta}{r}+\int_{\tan^{-1}(\frac{Y2}{X1})}^{\frac{\pi}{2}} \int_{\varepsilon}^{\frac{Y2}{\sin(\theta)}} \frac{drd\theta}{r}-\left((1-T_{b})\times\iint_{shieled parts} \frac{drd\theta}{r}\right) \end{matrix}\right)=\int_{0}^{2\pi} \int_{\varepsilon}^{R_{eq}} \frac{drd\theta}{r}$$

Integration with respect to **r** leads to natural logarithm as follows:

$$\left(\begin{matrix} \int_{0}^{\tan^{-1}(\frac{Y1}{X1})} \ln(\frac{X1}{\varepsilon\cos\theta})d\theta+\int_{\tan^{-1}(\frac{Y1}{X1})}^{\frac{\pi}{2}} \ln(\frac{Y1}{\varepsilon\sin\theta})d\theta+\int_{0}^{\tan^{-1}(\frac{Y1}{X2})} \ln(\frac{X2}{\varepsilon\cos\theta})d\theta+\int_{\tan^{-1}(\frac{Y1}{X2})}^{\frac{\pi}{2}} \ln(\frac{Y1}{\varepsilon\sin\theta})d\theta+\int_{0}^{\tan^{-1}(\frac{Y2}{X2})} \ln(\frac{X2}{\varepsilon\cos\theta})d\theta\\ +\int_{\tan^{-1}(\frac{Y2}{X2})}^{\frac{\pi}{2}} \ln(\frac{Y2}{\varepsilon\sin\theta})d\theta+\int_{0}^{\tan^{-1}(\frac{Y2}{X1})} \ln(\frac{X1}{\varepsilon\cos\theta})d\theta+\int_{\tan^{-1}(\frac{Y2}{X1})}^{\frac{\pi}{2}} \ln(\frac{Y2}{\varepsilon\sin\theta})d\theta-\left((1-T_{b})\times\iint_{shieled parts} \frac{drd\theta}{r}\right) \end{matrix}\right)=\int_{0}^{2\pi} \int_{\varepsilon}^{R_{eq}} \frac{drd\theta}{r}$$

In the next step, integration respect to θ and by more simplification, it will be rearranged as Eq.2.

The algorithm to calculate profile was as follows:

1. PDDs were measured for a number of square fields along the central beam axis, for open fields and 45º wedge and for both 6 and 18 MV energies separately. The PDDs were tabulated for desired interval (e.g., 0.5cm) and stored in the MATLAB program.
2. A set of required parameters such as field size, wedge angle and blocks properties (e.g., thickness, location and size), was fed to the calculation algorithm
3. For open symmetric field, the equivalent square,$S_{eq(x_{0},0)}$, was calculated for any point placed on cross-line (x_0_, 0) using Eq.4.
4. To plot the profile at depth 10cm, PDD ($S_{eq(x_{0},0)}$) was interpolated from stored data for both energies at depth 10cm.
5. According Eq.5, 6, profiles were calculated for a series of estimated correction factors for a standard field 10cm by 10cm in depth 10 cm and both energies 6, 18MV
6. The γ-index was calculated for every point. σ_in_, σ_out_, T_J_ and CF_e.diseq_ were changed till$\gamma\leq1$.
7. Finally, CF_J_, CF_w_ and CF_b_ were calculated to plot profiles for asymmetric, wedged and irregular fields at any depth.
